# Hormonal Contraceptives and the Gut Microbiome in Female Athletes: Implications for Health, Performance, and Exercise-Related Physiological Adjustments

**DOI:** 10.7759/cureus.88789

**Published:** 2025-07-26

**Authors:** Weronika Pierudzka, Józef Slawatycki, Paula Klemenska, Konrad Warczak, Paulina Wasilewska, Paulina Horwat, Jan Górski

**Affiliations:** 1 Hospital Medicine, Poznan University of Medical Sciences, Poznań, POL; 2 Hospital Medicine, Jan Biziel University Hospital No. 2, Bydgoszcz, POL; 3 Hospital Medicine, District Public Hospital, Juraszów, POL

**Keywords:** athletic performance, exercise adaptation, female athletes, gut microbiome, hormonal contraceptives, physical exercise

## Abstract

Hormonal contraceptives are widely used among female athletes, with documented prevalence rates varying from 29% to 65% across different sports disciplines. Growing evidence suggests that hormonal contraceptives may influence the diversity and composition of the gut microbiome. A particular concern is a potential reduction in beneficial bacteria responsible for producing short-chain fatty acids, which could impact both overall health and the body's adaptation to exercise training. This narrative review examines the current understanding of the links between hormonal contraceptive use, changes in gut bacteria, and exercise-related physiological adaptations in female athletes. A comprehensive literature search of PubMed and Google Scholar (2017-2025) was conducted using terms related to hormonal contraception, gut microbiota, and exercise. Due to the scarcity of studies directly investigating all three factors together, the search also included research on the relationships between each pair of these elements. Eighty-eight relevant articles were identified and included. While regular physical activity is generally known to support a healthy and diverse gut microbiome, hormonal contraception might counteract these positive effects. Hormonal contraceptives appear capable of disrupting the community of gut microbes involved in regulating estrogen, potentially reducing overall microbial diversity and promoting harmful imbalances. Critically, within the scope of this review, only one pilot study has been identified as directly exploring these interactions in female athletes. This key study found that athletes using hormonal contraceptives showed a decrease in the types of gut bacteria that produce beneficial short-chain fatty acids. Proposed pathways through which contraceptive-induced microbial changes might affect exercise adaptation and performance include alterations in energy metabolism, impaired immune function, weakening of the gut barrier, and disruption of natural hormonal balance. The impact likely depends on the specific type of contraceptive used, with combined oral contraceptives potentially having stronger effects than progestin-only methods. Given the current limited evidence, particularly the lack of direct studies beyond the pilot study mentioned, it is premature to draw definitive causal conclusions. However, the potential interactions suggest important practical implications. These include developing individualized approaches to training programming, nutritional strategies (such as optimizing fiber and prebiotic intake to support beneficial microbes), and contraceptive counseling for female athletes (e.g., involving physicians, sports dietitians, and coaches). Future research must prioritize well-designed studies, including randomized controlled trials and long-term observations, specifically in athletic populations. Such research is essential to develop evidence-based guidelines that can help optimize both the health and performance of female athletes who use hormonal contraception. To our knowledge, this review is the first to synthesize the complex relationship between contraceptive use, gut bacteria, and exercise adaptation specifically within female athletic cohorts.

## Introduction and background

The human gut microbiota and its metabolites are fundamental regulators of physiological functions critical for human health and athletic performance. These include immune response modulation, energy metabolism, and cognitive function [1]. Within female physiology, emerging research highlights that natural fluctuations in sex hormones, particularly estrogen, exert a profound influence on the composition and functional capacity of the gut microbial community. Central to this interaction is the estrobolome, the specific subset of gut microbes capable of metabolizing and modulating bioactive estrogen levels. The estrobolome acts as a key bidirectional interface: it is regulated by host estrogen levels while simultaneously influencing systemic estrogen availability through enzymatic processes [2-4]. Consequently, these dynamics between sex hormones and the gut microbiota hold significant implications for metabolic health and athletic performance pathways. 

**Figure 1:**
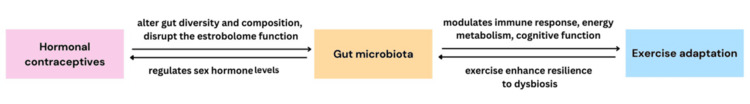
Conceptual framework of the tripartite relationship between hormonal contraceptives, gut microbiota, and exercise adaptation in female athletes

Hormonal contraceptives (HCs), by deliberately modifying endogenous estrogen and progesterone profiles, represent a potent external factor capable of disrupting this host-microbe dialogue. By altering the hormonal milieu that shapes the gut ecosystem, HCs may exert substantial effects on gut microbiota composition, diversity, and function, including the activity of the estrobolome [2,5-7]. While investigations have yielded inconsistent findings on the specific microbial taxa affected and the functional outcomes, the potential for HCs to induce gut microbial alterations, including states of dysbiosis (an imbalance or maladaptation in the gut microbial community structure and function), is a critical consideration. Such alterations could impact the microbiota's core roles in immune function, energy metabolism, and barrier integrity. This relationship is particularly important for female athletes. Their pursuit of peak performance and recovery hinges on the optimal function of physiological systems heavily influenced by the gut microbiota. Crucially, physical activity itself is a recognized modulator of gut microbiota composition, generally promoting greater microbial diversity and enhancing resilience against dysbiosis [8-10]. This suggests that the regular exercise inherent to an athlete's lifestyle may buffer against external perturbations to the gut microbiota, potentially counteracting some effects induced by HC use [11,12]. To conceptualize this tripartite relationship, Figure 1 presents a framework linking HCs, gut microbiota alterations, and exercise adaptation pathways.

Despite this, the complex interplay between HC-induced modulation of the gut microbiota, the physiological functions governed by the microbiota, and exercise adaptations remains poorly characterized. Therefore, the purpose of this review is to explore the hypothesis that HC use can influence exercise adaptation and performance in women through alterations in gut microbiota composition and function. We critically evaluate the evidence linking HC use to microbial changes, the impact of these microbial changes on exercise-relevant physiology, and the potential for exercise to mitigate HC-related microbial disruptions. To our knowledge, this is the first review to synthesize the relationship among HC use, gut microbiota composition, and exercise-related physiological changes in female athletes.

## Review

Study design

A comprehensive literature search was conducted using PubMed and Google Scholar, covering publications from 2017 to 2025, with a particular emphasis on studies published between 2020 and 2025. PubMed was chosen for its extensive biomedical indexing, while Google Scholar was used as a supplementary source to capture interdisciplinary literature that might not be available through PubMed. Google Scholar search was limited to the first 10 pages of the results, sorted by relevance. The search strategy employed Boolean combinations of the following keyword groups: [‘hormonal contraception’ OR ‘hormonal birth control’ OR ‘combined oral contraceptives’ OR ‘synthetic estradiol’] AND/OR [‚gut microbiome’ OR ‘gut microbiota’ OR ‘gut bacteria’] AND/OR [‚athletes’ OR ‘exercise’ OR ‘physical activity’]. The initial queries that combined all three domains yielded only one directly relevant study, thus highlighting a substantial gap in the literature. To address this, the search strategy was intentionally broadened to include studies investigating bilateral relationships, specifically (1) hormonal contraception and gut microbiota, (2) gut microbiota and exercise adaptation, and (3) hormonal contraception and physical performance. To further minimize the risk of omission, the reference lists of all eligible articles were manually reviewed to identify additional relevant studies. Studies were included if they met the following criteria: peer-reviewed original research articles, reviews, scoping reviews, systematic reviews, or meta-analyses; investigations involving human subjects, preferably female athletes or physically active women; and articles addressing at least two of the three focal domains, that is, hormonal contraception, gut microbiota, or exercise-related outcomes. Only English-language publications dated from 2017 onward were considered. The exclusion criteria were case reports, articles published before 2017, non-English publications, studies conducted exclusively on animals, and articles limited to a single domain without relevance to the others. A total of 88 articles met the inclusion criteria. The screening process was conducted independently by four reviewers, who assessed titles, abstracts, and full texts. Discrepancies in study inclusion were resolved through group discussion and consensus. Due to significant heterogeneity in study populations, interventions, and outcome measures, no quantitative synthesis or meta-analysis was feasible. Instead, findings were synthesized narratively to explore emerging conceptual patterns, proposed mechanistic pathways, and current gaps in the evidence base.

HCs in female athletes

Classification and Mechanisms of Action

HCs constitute a pharmacologically diverse group primarily employed to prevent pregnancy via the suppression of ovulation and modulation of the reproductive hormonal environment through synthetic estrogens and/or progestins. Female athletes commonly utilize two main methods: combined hormonal contraceptives (CHCs) and progestin-only methods. CHCs contain both estrogen (typically ethinylestradiol) and progestin components, with various formulations differing in hormone dosage and progestin type. Progestin-only methods include progestin-only pills (POPs) and long-acting reversible contraceptives (LARCs) such as subdermal implants, hormonal intrauterine systems (IUS), and injectable formulations [13]. 

Prevalence and Usage Patterns Among Female Athletes

The global prevalence of HC use among female athletes ranges from 29% to 65%, with lifetime use approaching 70% in some cohorts [13-17]. Among the 476 elite athletes, combined oral contraceptives (COCs) were the predominant choice, accounting for 68.5% of HC use, while progestin-only methods comprised 30% [13]. Sport-specific patterns have emerged, with progestin-only formulations showing higher prevalence in endurance sports, potentially reflecting concerns about the cardiovascular and metabolic effects of ethinylestradiol [18]. Interestingly, a substantial proportion of elite athletes (54%), particularly in lean sports, avoid HCs entirely due to concerns often linked to energy deficiency, opting for non-hormonal or no contraception [19].

Menstrual Regulation and Therapeutic Uses in Athletes

Female athletes utilize HCs for various reasons beyond pregnancy prevention, including menstrual cycle regulation or manipulation (e.g., scheduling withdrawal bleeds, achieving amenorrhea), mitigation of menstrual-related symptoms (dysmenorrhea, premenstrual syndrome), and management of conditions such as acne, polycystic ovary syndrome (PCOS), or endometriosis [13]. While strategic cycle management for training or competition is a potential consideration, current evidence suggests this practice is not yet widespread [16].

Systemic Physiological Effects Relevant to Sport

The physiological impact of HCs extends significantly beyond reproductive function, influencing critical athletic performance parameters including metabolism, adaptation, and recovery. Synthetic hormones, particularly in CHCs, suppress the hypothalamic-pituitary-ovarian axis and reduce endogenous estrogen and progesterone levels [20]. This altered endocrine profile modulates multiple physiological systems relevant to exercise performance, including substrate utilization, muscle adaptation, vascular function, and bone metabolism [21,22]. 

The gut microbiota: a key modulator of host physiology

Taxonomic Composition and Sex-Related Variability

The human gut microbiota represents a complex ecosystem of trillions of microorganisms, including bacteria, fungi, and viruses, that maintain symbiotic relationships with the host, critically regulating metabolic, endocrine, and immune functions [23,24]. This microbial community is predominantly structured around two core bacterial phyla, *Firmicutes* and *Bacteroidetes*, which collectively constitute approximately 90% of intestinal bacteria, alongside less abundant phyla including *Actinobacteria*, *Verrucomicrobia*, and *Proteobacteria* [25]. *Firmicutes* comprise Gram-positive, facultative anaerobes notable for spore formation. This phylum is primarily represented by genera such as *Clostridium*, *Lactobacillus*, *Faecalibacterium*, *Ruminococcus*, and *Enterococcus*. In contrast, *Bacteroidetes* are Gram-negative, non-sporulating anaerobes primarily represented by *Bacteroides* and *Prevotella* [12]. The composition and functional profiles of the gut microbiota exhibit significant sex-related differences, with Zhang et al. demonstrating distinct trajectories in the microbial community structure between males and females across their lifespan [11]. These sex-specific patterns are particularly relevant when considering the impact of sex hormones and HCs on microbial ecosystems in female athletes [26]. Among the minor phyla, *Actinobacteria* features the genus *Bifidobacterium*, which enhances gut barrier integrity, facilitates carbohydrate metabolism, and modulates immune responses [27]. *Verrucomicrobia* are epitomized by *Akkermansia muciniphila*, a mucin-degrader critical for mucosal layer maintenance and metabolic regulation [28], and *Proteobacteria* include opportunistic pathogens (e.g., *Escherichia, Salmonella*, and *Helicobacter*) that may induce dysbiosis when microbial balance is disrupted [29]. Additionally, specific bacterial families such as *Enterobacteriaceae* play important ecological roles in gut health and disease, influencing host immune responses and metabolic functions through complex interactions with other microbial communities [29].

Functional Roles in Metabolism and Immune Regulation

Functionally, gut microorganisms drive essential metabolic processes through dietary fiber fermentation, producing short-chain fatty acids (SCFAs), primarily acetate, propionate, and butyrate. *Bacteroidetes* predominantly generate acetate and propionate, whereas *Firmicutes* are primary butyrate producers [24]. SCFAs serve as primary energy substrates for intestinal epithelial cells, influence glucose and lipid metabolism, modulate hepatic fat storage, and impact the gut-brain axis, all of which are essential for maintaining energy homeostasis and supporting athletic performance [26]. Beyond their role as energy substrates, SCFAs function as signaling molecules, affecting neuroendocrine-immune networks through multiple mechanisms: (1) activation of G protein-coupled receptors (GPR41, GPR43, GPR109A); (2) inhibition of histone deacetylases, modulating gene expression; (3) regulation of host epigenetics; and (4) protection against pathogenic microbial infections [30]. The role of SCFAs in promoting exercise adaptation will be examined in greater detail subsequently.

Gut Barrier Integrity and Host Protection

The gut microbiota is also crucial for regulating gut barrier integrity and influencing intestinal permeability [31]. The intestinal barrier functions as a selective filter, allowing the absorption of nutrients while excluding harmful stimuli. Disruptions in gut microbiota composition can increase intestinal permeability, leading to conditions such as "leaky gut syndrome", which promotes systemic inflammation and tissue damage [31]. Recent studies indicate potential benefits of specific bacterial species, such as *Akkermansia* *muciniphila*-derived extracellular vesicles (AmEVs), which enhance tight junction integrity, reduce body weight gain, and improve glucose tolerance, potentially benefiting women athletes by managing metabolic health and performance [32]. 

Microbial Diversity, Modulating Factors, and Athletic Adaptation

The composition of the gut microbiota is characterized by two primary diversity metrics: alpha diversity, which refers to the richness (number of species) and evenness (relative abundance distribution) of species within a single sample, and beta diversity, which measures the differences in microbial composition between samples [33]. Higher alpha diversity is generally associated with better health outcomes, whereas beta diversity can reflect the impact of environmental factors or interventions on microbial communities [33]. Various factors influence the diversity and function of gut microbiota, including diet, age, birth method, antibiotic exposure, probiotics use, and physical activity [33]. High-fiber diets specifically promote SCFA-producing bacterial populations, while probiotics such as *Lactobacillus* strains modulate gut microbiota composition, potentially alleviating metabolic disorders [34].

For athletes, the gut microbial community functions as a vital metabolic organ that optimizes nutrient absorption, vitamin synthesis, and energy harvest while modulating immune homeostasis through toll-like receptor (TLR) signaling cascades [35].

Impact of HCs on the gut microbiota

Microbiota-Hormone Crosstalk and Evidence from Human Studies

The interaction between hormones and gut microbiota constitutes a complex, bidirectional endocrine-microbiome dialogue, which holds significant implications for women's health and potentially for athletic performance as well [2,3]. This multifaceted interaction operates through two principal physiological pathways. The first pathway involves the gut microbiota's influence on hormonal regulation via the estrobolome, a specialized functional consortium comprising primarily *Firmicutes*, *Bacteroidetes*, and *Proteobacteria* phyla [36]. These microbial communities encode specific enzymatic activities, notably β-glucuronidases and β-glucosidases, which facilitate the deconjugation of estrogen metabolites, thereby modulating circulating hormone levels and establishing a complex microbial-endocrine feedback regulatory system [36]. The second pathway encompasses the reciprocal impact of exogenous hormonal compounds on microbial ecosystem composition and functionality through various physiological mechanisms that alter the intestinal environment [37]. This bidirectional relationship creates a dynamic system where HCs may induce changes in gut microbiota composition, which in turn may affect hormonal metabolism and signaling, with potential consequences for physiological functions relevant to exercise performance. 

Current evidence from human studies reveals context-dependent effects of HC on gut microbiota, with methodological variations contributing to inconsistent findings across the literature. Mihajlovic et al. observed reduced α-diversity in healthy premenopausal COC users, characterized by decreased abundance of *Eubacterium *(p=3.96×10^−15^), *Haemophilus *(p=0.0006), and unclassified *Firmicutes* (p=3.79×10^−7^), alongside enrichment of *Akkermansia* (p=0.0114) and *Barnesiella *(p=7.31×10^−9^) [2]. Conversely, a longitudinal study by Hua et al. reported no significant alterations in overall microbial diversity following HC initiation. However, they documented a temporal increase in the relative abundance of *Actinobacteria* and *Firmicutes* phyla, concurrent with a decline in *Bacteroidetes* phylum and specific species, including *Clostridium* *asparagiforme*, *Clostridium* *hathewayi*, *Clostridium* *symbiosum*, and *Eubacterium* *eligens*. Notably, Hua et al. identified an association between sex hormone levels and shifts in microbiome composition and function post-HC use [7]. Terrazas et al. also found no significant changes in either α- or β-diversity associated with HC use. Nevertheless, their analysis (p<0.05) indicated a higher relative abundance of *Lachnospiraceae*, *Barnesiella*, *Faecalibacterium*, and *Clostridium* species among COC users compared to non-users. Non-users, conversely, exhibited a higher relative abundance of *Eisenbergiella* bacterium, *Dorea* sp., and *Alistipes* sp. These compositional differences were potentially linked to hormonal fluctuations [5].

Importantly, a pilot study by Brito et al. is the only one to date to directly investigate physically active women. This study reported altered β-diversity (p=0.015) and a reduced abundance of several SCFA-producing bacterial taxa among HC users (unadjusted p≤0.046), raising concerns about the metabolic implications of these shifts in athletic populations [6].

A summary of key findings from these studies, including the types of HCs used, microbiota profiling methods, observed diversity changes, compositional alterations, and impacts on SCFA-producing taxa, is presented in Table 1. 

**Table 1:** Summary of studies investigating the impact of HC use on gut microbiota HC: hormonal contraceptive; COCs: combined oral contraceptives; OCs: oral contraceptives; SCFA: short-chain fatty acid; 16S rRNA: 16S ribosomal RNA Only taxa with a significant change in abundance were included in the table.

Author (year)	Study design	Population	HC use duration	HC type	Microbiota analysis method	Diversity changes following HC use	Compositional changes following HC use	Changes in SCFA-producing taxa
Mihajlovic et al. (2021) [2]	Observational study	General female population	Not specified	COCs	16S rRNA sequencing	Reduced α-diversity	↑: *Akkermansia* (p=0.0114), *Barnesiella* (p=7.31×10^−9^). ↓: *Eubacterium* (p=3.96×10^−15^), *Haemophilus* (p=0.0006), *Firmicutes* unclassified (p=3.79×10^−7^)	Yes
Hua et al. (2022) [7]	Observational study	General female population	6 months	OCs	Shotgun metagenomics	No significant change	↑: *Actinobacteria*, *Firmicutes*. ↓: *Bacteroidetes*, *Clostridium asparagiforme*, *Clostridium hathewayi*, *Clostridium symbiosum*, *Eubacterium eligens*	Yes
Terrazas et al. (2025) [5]	Observational study	General female population	1-5 years	COCs	16S rRNA sequencing	No significant change	↑: *Lachnospiraceae*, *Barnesiella*, *Faecalibacterium*, *Clostridium* sp. (p<0.05). ↓: *Eisenbergiella*, *Dorea *sp., *Alistipes *sp. (p<0.05)	Yes
Brito et al. (2025) [6]	Observational study	Physically active women	7 months-7.7 years	Mixed HC types	16S rRNA sequencing	Altered β-diversity	↓: *Alistipes shahii*, *Dorea longicatena*, *Gemmiger* sp003476825, *Blautia massiliensis*, *Eubacterium hallii*, *Blautia obeum*, *Faecalibacterium prausnitzii* (unadjusted p≤0.046)	Yes

Mechanisms Underlying HC-Induced Microbial Shifts

The potential mechanisms underlying HC-induced microbial shifts are multifaceted and include (1) disruption of estrobolome function through the suppression of ovarian estrogen production and introduction of synthetic hormones [38]; (2) alterations in bile acid metabolism and gastrointestinal motility [39]; and (3) immunomodulation of gut-associated lymphoid tissue (GALT), potentially precipitating a shift toward pro-inflammatory immunological states [40].

Functionally, reductions in SCFA-producing taxa may compromise energy supply to colonocytes, modulate immune function, impair gut barrier integrity, and disrupt systemic metabolism [26]. These changes are accompanied by alterations in the *Firmicutes*-to-*Bacteroidetes* ratio and microbial functional pathways, including carbohydrate metabolism, amino acid biosynthesis, and xenobiotic degradation, indicating broad physiological implications [41]. For athletic populations, where exercise generally induces beneficial microbial adaptations, HC-associated dysbiosis may antagonize these adaptations by disrupting energy metabolism, immune homeostasis, inflammation control, and recovery efficiency, thereby compromising the marginal physiological advantages essential for peak performance [42].

Gut microbiota and exercise-related physiological adjustments

Bidirectional Relationship Between Exercise and Gut Microbiota

A growing body of evidence indicates the critical role of gut microbiota in exercise adaptation and performance [1,9,12,41-45]. This relationship is bidirectional: physical activity shapes the composition, diversity, and metabolic output of the gut microbiome, whereas microbial communities influence physiological responses to training through multiple mechanisms [46]. The composition of gut bacteria affects various factors essential for athletic performance, including nutrient absorption efficiency, immune function, inflammation control, and cognitive function via the gut-brain axis [12]. This mutual interaction positions the gut microbiota as both a target and a mediator of exercise-related physiological adaptations.

Exercise-Induced Changes in Microbiota Composition

Regular physical activity has been shown to beneficially shape the gut microbiota. Research indicates that athletes typically exhibit higher microbial diversity compared to sedentary individuals, a factor contributing to overall gut health [47-49]. Their gut microbiota is often enriched with bacterial species involved in the metabolism of amino acids, carbohydrates, and fiber [48]. This metabolic activity facilitates the production of SCFAs. Notably, this enrichment of SCFA-producing taxa was predominantly observed within butyrate-producing lineages from the *Firmicutes* phylum, such as *Eubacterium rectale*, *Eubacterium* *hallii*, *Roseburia* *hominis* (*Lachnospiraceae*), and *Faecalibacterium* *prausnitzii* (*Ruminococcaceae*) [1,50]. Furthermore, *Faecalibacterium* *prausnitzii* synthesizes several bioactive compounds, including shikimic acid, salicylic acid, and the microbial anti-inflammatory molecule (MAM), which contribute to the maintenance of immune system homeostasis and the reduction of inflammatory responses [51]. Additionally, the *Verrucomicrobia* phylum, particularly *Akkermansia muciniphila*, is also more abundant in physically active individuals and has been associated with improved mucosal barrier integrity, enhanced lipid metabolism, and better insulin sensitivity [50].

Crucially, exercise-induced microbial responses are dictated by activity type, intensity, and timing. Endurance training robustly increases *Akkermansia* abundance [52], while high-intensity exercise stimulates butyrate- and succinate-producing bacteria, enhancing microbial homeostasis and immune modulation [45]. This relationship is intensity-dependent: low-to-moderate exercise generally boosts microbial diversity and functional resilience, whereas prolonged high-intensity exertion can compromise intestinal barrier function (inducing "leaky gut") and trigger transient dysbiosis [8]. Nevertheless, elite athletes often develop compensatory microbial adaptations that preserve gut integrity under extreme training loads [45].

Further nuance emerges from studies on training periodization. Akazawa et al. demonstrated that distinct phases of structured training dynamically alter gut microbiota in elite athletes [53]. During preparation phases, athletes exhibited elevated *Prevotella*, *Bifidobacterium*, *Parabacteroides*, and *Alistipes* compared to transition phases. These microbial shifts correlated significantly with aerobic capacity and trended toward association with anaerobic power, suggesting the microbiota may actively support physiological adaptation to exercise-specific demands [53].

Functional Role of the Microbiota in Exercise Adaptation

Beyond compositional shifts, the gut microbiota supports exercise performance and recovery through a variety of functional mechanisms. SCFAs, produced by the fermentation of dietary fiber, play critical roles in host energy metabolism [54]. These metabolites not only fuel colonocytes but also act as systemic signaling molecules that stimulate muscle protein synthesis, enhance mitochondrial biogenesis, and regulate substrate utilization, thus facilitating metabolic adaptations to exercise [55]. In addition, gut microbes help neutralize reactive oxygen species generated during high-intensity physical activity, thereby supporting antioxidant capacity and accelerating tissue recovery [56]. They also modulate immune responses through both local and systemic pathways, contributing to the resolution of inflammation and the repair of exercise-induced microtrauma [40]. Perhaps most critically, the gut microbiota helps maintain intestinal barrier integrity, which is often challenged during prolonged or intense training due to splanchnic hypoperfusion, mechanical strain, or thermal stress [31]. A healthy microbiota protects against exercise-induced gut permeability by supporting tight junction proteins and promoting mucin production, thereby reducing the risk of systemic inflammation triggered by endotoxin translocation [57].

The tripartite interaction: HCs, gut microbiota, and exercise adaptations

Conceptual Framework of the Tripartite Interaction

The complex interrelationships between HCs, gut microbiota, and exercise adaptations form a dynamic tripartite interaction with significant implications for female athletes' health and performance. This interaction can be conceptualized as a bidirectional network where each component influences and is influenced by the others through multiple mechanistic pathways [3]. Simultaneously, both HCs and the gut microbiota independently and synergistically affect exercise adaptation processes, including energy metabolism, muscle protein synthesis, inflammation regulation, and recovery capacity [3,8,20,34,58].

The evidence supporting this tripartite model emerges from three established bilateral relationships: (1) HCs and gut microbiota interactions, characterized by alterations in microbial diversity and composition [2,5-7,38,59]; (2) gut microbiota and exercise adaptation relationships, where microbial metabolites influence physiological responses to training [8-10,40,42,45,49,55,56,60,61]; and (3) direct effects of HCs on exercise physiology through hormonal modulation [13,16-18,20,62]. The integration of these bilateral relationships provides a framework for understanding how HC-induced alterations in gut microbiota may indirectly affect exercise adaptations in female athletes.

Evidence for HC-Induced Microbiota Alterations in Female Athletes

The current evidence investigating the interplay between HC use, gut microbiota, and exercise adaptation in female athletes remains limited and heterogeneous. To date, only one pilot study has directly addressed this relationship in physically active women. Brito et al. conducted a cross-sectional analysis comparing gut microbiota composition between HC users (n=12) and non-users (n=12) among physically active females [6]. Using 16S rRNA gene sequencing, they observed significant differences in β-diversity (between-sample diversity) but not α-diversity (within-sample diversity) between the two groups. Most notably, HC users exhibited reduced abundance of seven taxa associated with SCFA-production: *Alistipes shahii, Dorea longicatena, Gemmiger *sp003476825, *Blautia massiliensis, Eubacterium hallii, Blautia obeum*, and *Faecalibacterium* *prausnitzii* [6]. These findings suggest that HC use may selectively suppress beneficial SCFA-producing bacteria in physically active women, potentially affecting metabolic function and exercise adaptation. However, this single study has important limitations that warrant consideration. The cross-sectional design precludes causal inference, the small sample size limits statistical power, and the heterogeneity in HC formulations introduces potential confounding. Additionally, the study did not directly assess functional metabolic outcomes or exercise performance parameters, limiting conclusions about the physiological significance of the observed microbial alterations.

In contrast, most available evidence on HC-microbiome interactions comes from general female populations, with key findings summarized in Table 1. While collectively suggesting HC use may perturb gut microbial communities, these studies report inconsistent findings due to significant variability in design, methodology, and HC formulations. Consequently, generalizing results to athletic populations is premature.

Mechanistic Pathways Linking HC-Induced Microbiota Changes to Exercise Adaptation

The potential impact of HC-induced microbiota alterations on exercise adaptation can be understood through several interconnected mechanistic pathways.

SCFA production and energy metabolism: SCFAs, particularly butyrate, acetate, and propionate, serve as critical metabolic intermediaries between gut microbiota and host physiology. The observed reduction in SCFA-producing taxa in HC users may have significant implications for energy metabolism during exercise [2,5,6]. SCFAs contribute approximately 10% of daily caloric requirements and serve as preferred energy substrates for colonocytes, sparing glucose for skeletal muscle utilization during exercise [63]. Additionally, SCFAs regulate hepatic gluconeogenesis, adipose tissue lipolysis, and skeletal muscle glucose uptake through the activation of G protein-coupled receptors and inhibition of histone deacetylases [30].

In athletic contexts, reduced SCFA production may compromise metabolic flexibility, that is, the capacity to efficiently switch between carbohydrate and fat oxidation based on exercise intensity and duration [35]. This could potentially impair endurance performance, particularly during prolonged high- to moderate-intensity exercise where fat oxidation contributes significantly to energy production. Furthermore, butyrate specifically enhances mitochondrial biogenesis and function through peroxisome proliferator-activated receptor gamma coactivator 1-alpha (PGC-1α) activation, a key regulator of exercise-induced mitochondrial adaptations [55,64]. HC-associated reductions in butyrate-producing taxa may therefore attenuate mitochondrial adaptations to endurance training, although direct evidence in female athletes remains lacking [65].

Immune modulation and inflammatory responses: The gut microbiota can contribute to the regulation of systemic and local inflammation through interactions with GALT and production of immunomodulatory metabolites, including SCFAs, tryptophan metabolites, and bile acid metabolites [66]. These metabolites play a crucial role in maintaining both gut and systemic homeostasis by inhibiting inflammatory immune cells and facilitating the differentiation and function of immunosuppressive cells [67].

SCFA-producing bacteria, which appeared reduced in HC users, exert anti-inflammatory effects through multiple mechanisms: (1) activation of regulatory T cells via histone deacetylase inhibition; (2) suppression of pro-inflammatory cytokine production in macrophages and dendritic cells; and (3) maintenance of intestinal barrier integrity, preventing the translocation of inflammatory bacterial component [26,54,66]. Similarly, certain microorganisms in gut microbiota critically process dietary tryptophan into immunoregulatory metabolites, particularly indole derivatives (e.g., indole-3-aldehyde, indole-3-propionic acid) that act as ligands for the aryl hydrocarbon receptor (AhR) [68]. HC-associated dysbiosis may disrupt this microbial conversion. These microbially derived indoles are essential for maintaining intestinal barrier integrity, promoting anti-inflammatory responses, and stimulating protective IL-22 production, while suppressing pro-inflammatory cytokines, thereby calibrating the balance between immune tolerance and defense [67]. It is noteworthy that the production of specific tryptophan-derived metabolites is influenced not only by the prevalence of tryptophan-metabolizing bacteria but also by the substrate-dependent regulation of metabolic pathways. This suggests that variations in the abundance of fiber-degrading bacteria can affect the competition for tryptophan among other microbial populations, ultimately impacting the production of various metabolites [69]. Furthermore, specific bacteria, such as *Akkermansia muciniphila*, transform primary bile acids into secondary bile acid metabolites (e.g., lithocholic acid, deoxycholic acid) [70]. HC-induced dysbiosis may alter this transformation. Secondary bile acids influence immune function by acting as signaling molecules through receptors like the farnesoid X receptor (FXR) and the G protein-coupled bile acid receptor 1 (TGR5). They can promote the differentiation of anti-inflammatory regulatory T cells (Tregs) and suppress the differentiation of pro-inflammatory Th17 cells, contributing to immune homeostasis [67,71]. An HC-altered microbiome, potentially disrupting the production of secondary bile acids alongside SCFAs and beneficial tryptophan metabolites, could therefore impair multiple vital immunoregulatory axes.

Consequently, the reduced abundance of beneficial bacterial taxa and the resultant dysregulation of SCFA, tryptophan-derived indole, and secondary bile acid metabolite production may synergistically promote a pro-inflammatory state, potentially exacerbating exercise-induced inflammation [66,71]. This multifaceted immune dysregulation may be particularly relevant during periods of intensified training or competition, when athletes experience transient immunosuppression and increased susceptibility to upper respiratory tract infections [72]. HC-associated microbiota alterations could theoretically compound this "open window" of immunovulnerability by disrupting immunomodulatory pathways dependent on SCFAs, AhR ligands, and secondary bile acids. This may negatively affect the resolution of exercise-induced inflammatory responses and recovery processes, although clinical evidence specifically linking HC-microbiota-metabolite-immunity interactions to exercise adaptation in female athletes is currently lacking.

Gut barrier function and systemic endotoxemia: Intense exercise can compromise intestinal barrier integrity through splanchnic hypoperfusion and hyperthermia, leading to increased intestinal permeability and translocation of bacterial lipopolysaccharide (LPS) into circulation, a phenomenon termed "exercise-induced endotoxemia" [73]. This endotoxemia contributes to systemic inflammation, gastrointestinal distress, and potentially impaired recovery [57].

The gut microbiota's role in maintaining intestinal barrier function is facilitated through the production of SCFAs (particularly butyrate), which enhance tight junction protein expression and mucin production [26,31,57]. HC-induced reductions in SCFA-producing bacteria may compromise this protective function, potentially exacerbating exercise-induced intestinal permeability [31]. Paradoxically, the observed increase in *Akkermansia muciniphila* in some HC users may partially counteract this effect, as this species can alleviate inflammatory responses and help in maintaining intestinal barrier function through the stimulation of mucin production and enhancement of tight junction integrity [32]. However, an overabundance of *Akkermansia muciniphila* in the gut can lead to compromised gut barrier functions, through excessive mucin degradation, and elevated serum LPS levels [74].

Estrobolome function and hormonal homeostasis: The estrobolome represents a critical interface between HCs, gut microbiota, and exercise physiology [9]. This microbial community modulates estrogen bioavailability through the enzymatic deconjugation of estrogen metabolites, influencing circulating hormone levels and tissue exposure [36].

HC administration fundamentally disrupts estrobolome function through multiple mechanisms: (1) suppression of endogenous estrogen production, reducing substrate availability for microbial β-glucuronidases; (2) introduction of synthetic estrogens and progestins with structural differences that may alter microbial metabolism; and (3) direct effects on the abundance and activity of estrogen-metabolizing bacteria [36,38].

These alterations in estrobolome function may have significant implications for exercise adaptation in female athletes. Estrogen influences multiple aspects of exercise physiology, including substrate utilization (promoting fat oxidation), muscle protein synthesis, neuromuscular function, and tendon mechanical properties [21]. Disruption of estrogen homeostasis through HC-induced microbiota alterations could potentially affect these processes, although the specific mechanisms and magnitude of effect require further investigation.

Differential Effects of HC Types on Gut Microbiota and Exercise Adaptation

Although direct evidence remains scarce, especially in athlete-specific populations, it is plausible that the impact of HCs on the gut microbiota, and consequently on exercise adaptation, varies depending on the type, formulation, dosage, and route of administration. Existing studies suggest that COCs may exert more pronounced effects on gut microbial diversity and composition than progestin-only methods, possibly due to their dual hormonal action and greater suppression of endogenous hormone fluctuations [2]. However, these conclusions are drawn primarily from observational research in general female populations and should be interpreted with caution.

Within COC formulations, the specific progestin component may differentially affect gut microbiota due to variations in androgenic, glucocorticoid, and mineralocorticoid receptor binding affinities [75]. These differences may subtly alter host physiology (e.g., bile acid metabolism, immune signaling), potentially creating a microenvironment conducive to microbial shifts.

Similarly, the route of administration may modulate microbiome interactions. Non-oral contraceptives, such as transdermal patches, vaginal rings, or intrauterine systems, bypass hepatic first-pass metabolism, potentially resulting in different systemic hormone concentrations and bioactivity compared to oral methods [76]. This pharmacokinetic distinction could influence the gut ecosystem, but no studies to date have directly examined this effect.

To date, no research has specifically assessed the influence of progestin-only contraceptives on gut microbiota in either general or athletic populations. Nonetheless, given that the gut microbiota is sensitive to hormonal changes, it is reasonable to speculate that progestin-only methods could also exert some microbiome-modulating effects, albeit potentially milder than those observed with COCs.

These formulation-specific effects may carry practical implications for female athletes selecting contraceptive methods. Athletes particularly concerned about gut health and exercise adaptation might consider progestin-only or locally acting methods, which potentially exert less pronounced effects on gut microbiota. However, these theoretical considerations must be balanced against individual contraceptive needs, side effect profiles, and performance considerations.

Practical implications and personalized interventions for female athletes

Given the complex interplay between HC use, gut microbiota, and exercise adaptation, personalized approaches to contraceptive selection for female athletes are warranted. Athletes should carefully evaluate their contraceptive choices in consultation with healthcare providers, considering not only contraceptive efficacy and traditional side effects but also potential impacts on gut health and performance. When selecting HC formulations, progestin-only methods or locally acting HCs may theoretically exert less pronounced effects on gut microbiota than COCs, although direct comparative evidence is lacking [2]. 

Personalized nutritional interventions represent a promising avenue for mitigating potential microbiota-related disturbances and optimizing performance outcomes in female athletes using HCs. A diet enriched with prebiotic-rich foods such as fruits, vegetables, legumes, and whole grains can support SCFA production, essential for gut health and recovery [77]. Fermented foods like yogurt, kefir, sauerkraut, and kimchi may introduce beneficial microbial strains, while polyphenol-rich foods such as berries, dark chocolate, green tea, and olive oil could positively modulate gut microbiota composition [78]. Furthermore, specific nutritional compounds such as zinc-carnosine, glutamine, and collagen peptides may protect intestinal mucosa, enhance gut barrier integrity, and aid recovery following intensive exercise [79]. The role of dietary fiber and prebiotics in supporting gastrointestinal microbiota is particularly relevant for female athletes using HCs. Holscher has demonstrated that specific types of dietary fibers selectively promote the growth of beneficial bacterial populations, potentially counteracting HC-induced microbial alterations [80]. Similarly, Kaewarsar et al. have elucidated the mechanisms through which prebiotics modulate gut microbiota composition and function, offering targeted nutritional strategies for optimizing microbial health in female athletes [81]. Targeted supplementation strategies may also benefit female athletes. Selected probiotic strains, including *Lactobacillus acidophilus*, *Bifidobacterium* *lactis*, and *Lactobacillus* *plantarum*, have been shown to mitigate exercise-induced gastrointestinal symptoms, enhance gut barrier function, and reduce systemic inflammation [82,83]. Lee et al. specifically demonstrated that *Lactobacillus plantarum *TWK10 supplementation effectively attenuates exercise-induced oxidative stress and inflammation, which could be particularly beneficial for female athletes experiencing HC-induced alterations in gut microbiota [82]. Prebiotic supplements such as resistant starch, inulin, and galacto-oligosaccharides could further boost SCFA production, particularly beneficial when microbial populations are compromised [81]. Proper nutrient timing, as outlined in the International Society of Sports Nutrition position stand, is another important consideration for female athletes using HCs [84]. Strategic timing of nutrient intake around training sessions may help optimize substrate availability and recovery processes, potentially mitigating any metabolic disadvantages resulting from HC-induced alterations in gut microbiota and energy metabolism [84].

Training periodization should likewise be individualized, accounting for altered recovery capacities and adaptation responses related to HC-induced microbiome alterations [53]. Athletes and coaches should employ personalized monitoring protocols, incorporating both subjective measures (e.g., gastrointestinal symptom logs, perceived recovery scales) and objective performance assessments to refine training intensity, frequency, and recovery strategies [85]. Ultimately, optimizing athlete health and performance requires a collaborative, individualized approach integrating contraceptive needs, nutritional strategies, training modifications, and continuous monitoring of gut health [86]. 

Limitations

This review incorporates a broad range of study types, including observational studies, clinical trials, and previous reviews, which introduces variability in methodological quality and increases the risk of bias. A major limitation is the methodological and clinical heterogeneity across studies, which precluded formal statistical heterogeneity assessment. Included research varied in terms of HC formulations, administration routes, and duration of use. In addition, microbiome profiling methods ranged from 16S rRNA sequencing to shotgun metagenomics, affecting taxonomic and functional resolution and hindering direct comparison. Second, the limited representation of female athletes is a critical gap. Only one pilot study has specifically examined this population [6]. This limitation complicates the extrapolation of findings from the general populations, particularly when considering the unique physiological context associated with athletic training. Third, the predominance of observational studies limits causal inference and increases susceptibility to confounding factors. Key factors such as diet, sleep, stress, and training load were inconsistently reported or controlled, potentially biasing microbiome-related outcomes. Fourth, variation in outcome measures and lack of standardized exercise adaptation metrics (e.g., performance, recovery, physiological markers) limit the interpretation of functional implications of microbiome shifts. Finally, publication bias cannot be excluded, as studies reporting significant findings may be more likely to be published than null results, potentially skewing the evidence base. Despite these limitations, this review offers an important synthesis of early evidence and highlights the substantial knowledge gaps. Therefore, these findings should be interpreted with caution. Future research should prioritize randomized controlled trials, longitudinal cohort studies, and multi-omics approaches, with a focus on female athlete-specific populations, standardized protocols, and comprehensive control of confounding variables, to better elucidate the complex relationship between HC, gut microbiota, and exercise adaptation.

## Conclusions

This review underscores the complex and critically underexplored interaction between HC use, gut microbiota composition, and exercise adaptation in female athletes. While HC use is established to influence systemic physiology and alter gut microbial diversity and specific bacterial populations, and while the gut microbiome itself plays a crucial role in metabolic, immune, and performance functions relevant to training and recovery, the current body of evidence regarding their combined impact on athletic performance remains insufficient, inconsistent, and limited. Consequently, definitive conclusions regarding the performance implications of this interaction in female athletes cannot be drawn at present. Significant knowledge gaps persist, primarily due to methodological heterogeneity across existing studies, a scarcity of research specifically focused on female athletic populations, and the predominance of observational study designs. Nevertheless, the presented theoretical framework and preliminary findings highlight this interaction as a promising and vital area for future investigation. To definitively elucidate these intricate relationships and ultimately inform evidence-based guidelines for optimizing female athlete health and performance, future research must prioritize rigorous methodologies tailored to this specific question. This includes employing well-designed randomized controlled trials that carefully consider design choices (e.g., crossover vs. parallel-group) to account for individual variability and intervention carry-over effects. Studies should integrate targeted biomarker analysis relevant to the gut-exercise axis, such as SCFAs, zonulin (for gut permeability), and interleukin-6 (IL-6) (for inflammation), alongside performance metrics like VO₂max and sport-specific outcomes. Crucially, research must differentiate between athlete subpopulations, particularly comparing endurance (e.g., runners, cyclists) and strength/power athletes (e.g., sprinters, weightlifters), to account for divergent metabolic demands and training adaptations. Furthermore, such studies should utilize standardized protocols, employ advanced multi-omics approaches (metagenomics, metabolomics), comprehensively control for confounding variables (e.g., diet, training phase, specific HC formulation), and explore multi-site microbiome interactions beyond the gut. The reproductive microbiome, particularly sensitive to hormonal fluctuations induced by HCs, may interact with the gut microbiome in ways relevant to systemic inflammation, nutrient metabolism, and athletic performance. Understanding these crosstalk mechanisms represents an essential next step in mapping the full impact of HCs on female athletes. Addressing these priorities will bridge critical knowledge gaps and unravel the complex interplay between HC use, the gut microbiota, and exercise adaptation.

## References

[1] Varghese S, Rao S, Khattak A, Zamir F, Chaari A (2024). Physical exercise and the gut microbiome: a bidirectional relationship influencing health and performance. Nutrients.

[2] Mihajlovic J, Leutner M, Hausmann B (2021). Combined hormonal contraceptives are associated with minor changes in composition and diversity in gut microbiota of healthy women. Environ Microbiol.

[3] Leao L, Miri S, Hammami R (2025). Gut feeling: exploring the intertwined trilateral nexus of gut microbiota, sex hormones, and mental health. Front Neuroendocrinol.

[4] Schieren A, Koch S, Pecht T, Simon MC (2024). Impact of physiological fluctuations of sex hormones during the menstrual cycle on glucose metabolism and the gut microbiota. Exp Clin Endocrinol Diabetes.

[5] Terrazas F, Kelley ST, DeMasi T (2025). Influence of menstrual cycle and oral contraception on taxonomic composition and gas production in the gut microbiome. J Med Microbiol.

[6] Brito J, Grosicki GJ, Robinson AT (2025). Hormonal birth control is associated with altered gut microbiota β-diversity in physically active females across the menstrual cycle: a pilot trial. J Appl Physiol (1985).

[7] Hua X, Cao Y, Morgan DM, Miller K, Chin SM, Bellavance D, Khalili H (2022). Longitudinal analysis of the impact of oral contraceptive use on the gut microbiome. J Med Microbiol.

[8] Wegierska AE, Charitos IA, Topi S, Potenza MA, Montagnani M, Santacroce L (2022). The connection between physical exercise and gut microbiota: implications for competitive sports athletes. Sports Med.

[9] Clauss M, Gérard P, Mosca A, Leclerc M (2021). Interplay between exercise and gut microbiome in the context of human health and performance. Front Nutr.

[10] Ortiz-Alvarez L, Xu H, Martinez-Tellez B (2020). Influence of exercise on the human gut microbiota of healthy adults: a systematic review. Clin Transl Gastroenterol.

[11] Zhang Y, Diao R, Zhang L (2022). Effects of probiotics supplementation on the performance and metabolic health of overtraining athletes. J Food Nutr Res.

[12] Mohr AE, Jäger R, Carpenter KC (2020). The athletic gut microbiota. J Int Soc Sports Nutr.

[13] Martin D, Sale C, Cooper SB, Elliott-Sale KJ (2018). Period prevalence and perceived side effects of hormonal contraceptive use and the menstrual cycle in elite athletes. Int J Sports Physiol Perform.

[14] Nolan D, Elliott-Sale KJ, Egan B (2023). Prevalence of hormonal contraceptive use and reported side effects of the menstrual cycle and hormonal contraceptive use in powerlifting and rugby. Phys Sportsmed.

[15] Kirschbaum EM, Fischer K, Speiser D, Lautenbach F, Schwenkreis F, Dathan-Stumpf A, Legerlotz K (2025). Prevalence of menstrual dysfunction and hormonal contraceptive use among elite female athletes from different sports in Germany. Sports Med Open.

[16] Ekenros L, von Rosen P, Solli GS, Sandbakk Ø, Holmberg HC, Hirschberg AL, Fridén C (2022). Perceived impact of the menstrual cycle and hormonal contraceptives on physical exercise and performance in 1,086 athletes from 57 sports. Front Physiol.

[17] Oxfeldt M, Dalgaard LB, Jørgensen AA, Hansen M (2020). Hormonal contraceptive use, menstrual dysfunctions, and self-reported side effects in elite athletes in Denmark. Int J Sports Physiol Perform.

[18] Engseth TP, Andersson EP, Solli GS (2022). Prevalence and self-perceived experiences with the use of hormonal contraceptives among competitive female cross-country skiers and biathletes in Norway: the FENDURA project. Front Sports Act Living.

[19] Baumgartner S, Bitterlich N, Geboltsberger S, Neuenschwander M, Matter S, Stute P (2023). Contraception, female cycle disorders and injuries in Swiss female elite athletes-a cross sectional study. Front Physiol.

[20] Cabre HE, Joniak KE, Ladan AN (2024). Effects of hormonal contraception and the menstrual cycle on maximal strength and power performance. Med Sci Sports Exerc.

[21] Boisseau N, Isacco L (2022). Substrate metabolism during exercise: sexual dimorphism and women's specificities. Eur J Sport Sci.

[22] Williams JS, MacDonald MJ (2021). Influence of hormonal contraceptives on peripheral vascular function and structure in premenopausal females: a review. Am J Physiol Heart Circ Physiol.

[23] Novakovic M, Rout A, Kingsley T (2020). Role of gut microbiota in cardiovascular diseases. World J Cardiol.

[24] Portincasa P, Bonfrate L, Vacca M (2022). Gut microbiota and short chain fatty acids: implications in glucose homeostasis. Int J Mol Sci.

[25] Fujisaka S, Watanabe Y, Tobe K (2023). The gut microbiome: a core regulator of metabolism. J Endocrinol.

[26] Liu T, Sun Z, Yang Z, Qiao X (2023). Microbiota-derived short-chain fatty acids and modulation of host-derived peptides formation: focused on host defense peptides. Biomed Pharmacother.

[27] Gavzy SJ, Kensiski A, Lee ZL, Mongodin EF, Ma B, Bromberg JS (2023). Bifidobacterium mechanisms of immune modulation and tolerance. Gut Microbes.

[28] Mo C, Lou X, Xue J, Shi Z, Zhao Y, Wang F, Chen G (2024). The influence of Akkermansia muciniphila on intestinal barrier function. Gut Pathog.

[29] Moreira de Gouveia MI, Bernalier-Donadille A, Jubelin G (2024). Enterobacteriaceae in the human gut: dynamics and ecological roles in health and disease. Biology (Basel).

[30] Nshanian M, Gruber JJ, Geller BS (2025). Short-chain fatty acid metabolites propionate and butyrate are unique epigenetic regulatory elements linking diet, metabolism and gene expression. Nat Metab.

[31] Lama Tamang R, Juritsch AF, Ahmad R, Salomon JD, Dhawan P, Ramer-Tait AE, Singh AB (2023). The diet-microbiota axis: a key regulator of intestinal permeability in human health and disease. Tissue Barriers.

[32] Zhao S, Xiang J, Abedin M (2025). Characterization and anti-inflammatory effects of Akkermansia muciniphila-derived extracellular vesicles. Microorganisms.

[33] Hills RD Jr, Pontefract BA, Mishcon HR, Black CA, Sutton SC, Theberge CR (2019). Gut microbiome: profound implications for diet and disease. Nutrients.

[34] Hughes RL, Holscher HD (2021). Fueling gut microbes: a review of the interaction between diet, exercise, and the gut microbiota in athletes. Adv Nutr.

[35] Bongiovanni T, Yin MO, Heaney LM (2021). The athlete and gut microbiome: short-chain fatty acids as potential ergogenic aids for exercise and training. Int J Sports Med.

[36] Kumari N, Kumari R, Dua A (2024). From gut to hormones: unraveling the role of gut microbiota in (phyto)estrogen modulation in health and disease. Mol Nutr Food Res.

[37] Siddiqui R, Makhlouf Z, Alharbi AM, Alfahemi H, Khan NA (2022). The gut microbiome and female health. Biology (Basel).

[38] Zim A, Bommareddy A (2025). Estrogen-gut-brain axis: examining the role of combined oral contraceptives on mental health through their impact on the gut microbiome. Cureus.

[39] Zheng Z, Tang J, Hu Y, Zhang W (2022). Role of gut microbiota-derived signals in the regulation of gastrointestinal motility. Front Med (Lausanne).

[40] Cella V, Bimonte VM, Sabato C (2021). Nutrition and physical activity-induced changes in gut microbiota: possible implications for human health and athletic performance. Foods.

[41] Patel BK, Patel KH, Lee CN, Moochhala S (2024). Intestinal microbiota interventions to enhance athletic performance-a review. Int J Mol Sci.

[42] Marttinen M, Ala-Jaakkola R, Laitila A, Lehtinen MJ (2020). Gut microbiota, probiotics and physical performance in athletes and physically active individuals. Nutrients.

[43] Tarracchini C, Fontana F, Lugli GA (2022). Investigation of the ecological link between recurrent microbial human gut communities and physical activity. Microbiol Spectr.

[44] Donati Zeppa S, Agostini D, Gervasi M (2019). Mutual interactions among exercise, sport supplements and microbiota. Nutrients.

[45] Urban S, Chmura O, Wątor J, Panek P, Zapała B (2024). The intensive physical activity causes changes in the composition of gut and oral microbiota. Sci Rep.

[46] Bonomini-Gnutzmann R, Plaza-Díaz J, Jorquera-Aguilera C, Rodríguez-Rodríguez A, Rodríguez-Rodríguez F (2022). Effect of intensity and duration of exercise on gut microbiota in humans: a systematic review. Int J Environ Res Public Health.

[47] Munukka E, Ahtiainen JP, Puigbó P (2018). Six-week endurance exercise alters gut metagenome that is not reflected in systemic metabolism in over-weight women. Front Microbiol.

[48] Barton W, Penney NC, Cronin O (2018). The microbiome of professional athletes differs from that of more sedentary subjects in composition and particularly at the functional metabolic level. Gut.

[49] Humińska-Lisowska K, Zielińska K, Mieszkowski J (2024). Microbiome features associated with performance measures in athletic and non-athletic individuals: a case-control study. PLoS One.

[50] Bressa C, Bailén-Andrino M, Pérez-Santiago J (2017). Differences in gut microbiota profile between women with active lifestyle and sedentary women. PLoS One.

[51] Kurt EA, Demirci M, Ozbey D (2024). The role of Akkermansia muciniphila and Faecalibacterium prausnitzii in the pathogenesis of ulcerative colitis and Crohn's disease. Clin Lab.

[52] Hintikka JE, Ahtiainen JP, Permi P, Jalkanen S, Lehtonen M, Pekkala S (2023). Aerobic exercise training and gut microbiome-associated metabolic shifts in women with overweight: a multi-omic study. Sci Rep.

[53] Akazawa N, Nakamura M, Eda N (2023). Gut microbiota alternation with training periodization and physical fitness in Japanese elite athletes. Front Sports Act Living.

[54] Frampton J, Murphy KG, Frost G, Chambers ES (2020). Short-chain fatty acids as potential regulators of skeletal muscle metabolism and function. Nat Metab.

[55] Imdad S, Lim W, Kim JH, Kang C (2022). Intertwined relationship of mitochondrial metabolism, gut microbiome and exercise potential. Int J Mol Sci.

[56] Zhang L, Li H, Song Z, Liu Y, Zhang X (2024). Dietary strategies to improve exercise performance by modulating the gut microbiota. Foods.

[57] Dmytriv TR, Storey KB, Lushchak VI (2024). Intestinal barrier permeability: the influence of gut microbiota, nutrition, and exercise. Front Physiol.

[58] Aya V, Flórez A, Perez L, Ramírez JD (2021). Association between physical activity and changes in intestinal microbiota composition: a systematic review. PLoS One.

[59] Krog MC, Hugerth LW, Fransson E (2022). The healthy female microbiome across body sites: effect of hormonal contraceptives and the menstrual cycle. Hum Reprod.

[60] Lavilla-Lerma ML, Aibar-Almazán A, Martı Nez-Amat A, Benomar-El-Bakali N, Abriouel-Hayani H, Hita-Contreras F (2023). The effects of physical activity on the modulation of gut microbiota composition: a systematic review. Benef Microbes.

[61] Kulecka M, Fraczek B, Mikula M (2020). The composition and richness of the gut microbiota differentiate the top Polish endurance athletes from sedentary controls. Gut Microbes.

[62] Dasa MS, Kristoffersen M, Ersvær E (2021). The female menstrual cycles effect on strength and power parameters in high-level female team athletes. Front Physiol.

[63] Liu M, Lu Y, Xue G (2024). Role of short-chain fatty acids in host physiology. Animal Model Exp Med.

[64] Amiri P, Hosseini SA, Saghafi-Asl M, Roshanravan N, Tootoonchian M (2025). Expression of PGC-1α, PPAR-α and UCP1 genes, metabolic and anthropometric factors in response to sodium butyrate supplementation in patients with obesity: a triple-blind, randomized placebo-controlled clinical trial. Eur J Clin Nutr.

[65] Mozaffaritabar S, Koltai E, Zhou L (2024). PGC-1α activation boosts exercise-dependent cellular response in the skeletal muscle. J Physiol Biochem.

[66] Ney LM, Wipplinger M, Grossmann M, Engert N, Wegner VD, Mosig AS (2023). Short chain fatty acids: key regulators of the local and systemic immune response in inflammatory diseases and infections. Open Biol.

[67] Wang J, Zhu N, Su X, Gao Y, Yang R (2023). Gut-microbiota-derived metabolites maintain gut and systemic immune homeostasis. Cells.

[68] Kaur H, Bose C, Mande SS (2019). Tryptophan metabolism by gut microbiome and gut-brain-axis: an in silico analysis. Front Neurosci.

[69] Sinha AK, Laursen MF, Brinck JE (2024). Dietary fibre directs microbial tryptophan metabolism via metabolic interactions in the gut microbiota. Nat Microbiol.

[70] Nogal A, Louca P, Zhang X (2021). Circulating levels of the short-chain fatty acid acetate mediate the effect of the gut microbiome on visceral fat. Front Microbiol.

[71] Li C, Liang Y, Qiao Y (2022). Messengers from the gut: gut microbiota-derived metabolites on host regulation. Front Microbiol.

[72] Cicchella A, Stefanelli C, Massaro M (2021). Upper respiratory tract infections in sport and the immune system response. A review. Biology (Basel).

[73] Cinca-Morros S, Álvarez-Herms J (2024). The importance of maintaining and improving a healthy gut microbiota in athletes as a preventive strategy to improve heat tolerance and acclimatization. Microorganisms.

[74] Rajpurohit YS (2024). Dual-edged health benefit of Akkermansia muciniphila: impact on metformin and insulin resistance in type 2 diabetes - a perspective. Curr Top Diabetes.

[75] Krapf JM, Goldstein AT (2024). Combined estrogen-progestin oral contraceptives and female sexuality: an updated review. Sex Med Rev.

[76] Pant A, Maiti TK, Mahajan D, Das B (2023). Human gut microbiota and drug metabolism. Microb Ecol.

[77] Karačić A, Zonjić J, Stefanov E (2024). Short-term supplementation of sauerkraut induces favorable changes in the gut microbiota of active athletes: a proof-of-concept study. Nutrients.

[78] Balasubramanian R, Schneider E, Gunnigle E, Cotter PD, Cryan JF (2024). Fermented foods: harnessing their potential to modulate the microbiota-gut-brain axis for mental health. Neurosci Biobehav Rev.

[79] Wang L, Meng Q, Su CH (2024). From food supplements to functional foods: emerging perspectives on post-exercise recovery nutrition. Nutrients.

[80] Holscher HD (2017). Dietary fiber and prebiotics and the gastrointestinal microbiota. Gut Microbes.

[81] Kaewarsar E, Chaiyasut C, Lailerd N, Makhamrueang N, Peerajan S, Sirilun S (2023). Optimization of mixed inulin, fructooligosaccharides, and galactooligosaccharides as prebiotics for stimulation of probiotics growth and function. Foods.

[82] Lee MC, Hsu YJ, Chen MT (2024). Efficacy of Lactococcus lactis subsp. lactis LY-66 and Lactobacillus plantarum PL-02 in enhancing explosive strength and endurance: a randomized, double-blinded clinical trial. Nutrients.

[83] Di Dio M, Calella P, Pelullo CP, Liguori F, Di Onofrio V, Gallè F, Liguori G (2023). Effects of probiotic supplementation on sports performance and performance-related features in athletes: a systematic review. Int J Environ Res Public Health.

[84] Kerksick CM, Arent S, Schoenfeld BJ (2017). International society of sports nutrition position stand: nutrient timing. J Int Soc Sports Nutr.

[85] Jarrett H, Medlin S, Morehen JC (2025). The role of the gut microbiome and probiotics in sports performance: a narrative review update. Nutrients.

[86] Nolte S, Krüger K, Lenz C, Zentgraf K (2023). Optimizing the gut microbiota for individualized performance development in elite athletes. Biology (Basel).

